# Assessing the outcomes of HIV-infected persons receiving treatment for Kaposi sarcoma in Conakry-Guinea

**DOI:** 10.1186/s12885-017-3771-x

**Published:** 2017-12-02

**Authors:** Cavin E. Bekolo, Mohamed M. Soumah, Ousseni W. Tiemtore, Abdourahimi Diallo, Joseph-Desire Yuma, Letizia Di Stefano, Carol Metcalf, Mohamed Cisse

**Affiliations:** 1 Médecins Sans Frontières – Belgium (MSF-B), Conakry, Guinea; 2 Department of Dermatology and STD, Donka National Hospital, Conakry, Guinea; 3 Southern Africa Medical Unit (SAMU), Médecins Sans Frontières, Cape Town, South Africa

**Keywords:** HIV, Kaposi sarcoma, Antiretroviral therapy, Chemotherapy, Guinea

## Abstract

**Background:**

Médecins Sans Frontières is supporting comprehensive HIV care and treatment for Kaposi Sarcoma (KS) in Guinea, where antiretroviral coverage is low and access to KS treatment is very limited. We aimed to evaluate treatment response and survival outcomes of epidemic KS in this setting.

**Methods:**

Retrospective survival analysis of routinely collected clinical data of HIV-infected patients with clinically diagnosed KS, receiving ART and chemotherapy consisting of a combination of bleomycin and vincristine at the Donka National Hospital in Conakry between 2012 and 2015.

**Results:**

A total of 225 patients were enrolled for KS treatment within the three-year period. Late presentation with stage T1 disease was common (82.7%). At the end of a median of 8 cycles of chemotherapy (IQR: 2–12), complete remission was observed in 65 (28.9%), partial remission in 53 (23.6%), stable disease in 15 (6.7%) and unknown response for all 92 (40.9%) patients who dropped out of care. The chances of achieving complete remission doubled after each additional cycle of chemotherapy (aOR = 2.09 95% CI: 1.44–3.01) but were reduced by about two-thirds for each additional month delay between treatment and onset of KS (aOR = 0.31, 95% CI: 0.11–0.86). Treatment response was seriously compromised in patients with woody skin oedema (aOR = 0.05, 95% CI: 0.01–0.38) and those with prior chemotherapy (aOR = 0.21, 95% CI: 0.05–0.80). The median survival time was 7.6 months (95% CI: 5.9–9.8). Attrition from care was reduced by 22% for every additional cycle of chemotherapy administered (aH0R = 0.78, 95% CI: 0.71–0.84) and was lower in those with complete remission compared with those with partial or no response (aHR = 0.05, 95% CI: 0.007–0.43).

**Conclusion:**

There has been an increased access to KS treatment. The overall response rate is 52.4%, which is considered a satisfactory result. Poor outcomes were common and were largely due to late presentation and defaulting on treatment. Efforts towards early HIV/KS diagnosis and adherence to a full round of chemotherapy are needed for optimising outcomes. Newer drugs may be required for patients previously exposed to chemotherapy.

**Electronic supplementary material:**

The online version of this article (10.1186/s12885-017-3771-x) contains supplementary material, which is available to authorized users.

## Background

Epidemic Kaposi sarcoma (KS) is the most common neoplasm in persons living with the human immunodeficiency virus (HIV) worldwide. Sub-Saharan Africa (SSA) with a high burden of both HIV and human herpesvirus-8 (HHV-8) infections coupled with a more limited access to antiretroviral therapy (ART), still witnesses a high incidence of KS [1, 2]. Treatment response and survival outcomes also remain challenging in SSA. In a large community-based sample of patients diagnosed with KS in SSA, almost half became lost to follow-up by two years [3]. However, excellent clinical outcomes and retention in care have also been reported especially with the use of newer chemotherapeutic agents in combination with ART [4, 5].

The prevalence of human immunodeficiency virus (HIV) in Guinea remains low at approximately 1.6% in 2014 but treatment coverage also remains among the lowest in the world, with less than one in four (23%) people living with HIV accessing antiretroviral therapy (ART) [6, 7]. Voluntary HIV testing that aims for early diagnosis also remains low at approximately 4.8%. Consequently, about 82% of HIV-infected persons diagnosed in healthcare facilities are already in advanced stages of the disease [8, 9]. The burden of acquired immune deficiency syndrome (AIDS)-defining diseases like Kaposi sarcoma (KS) and cryptococcal meningitis remains alarmingly high in Guinea despite recent strides made by the country against HIV/AIDS [8, 10].

Kaposi sarcoma is the third major cause of hospital admissions and the leading cause of death at the Dermatology Unit of Donka National Hospital (DNH) in Conakry [11]. Even though antiretroviral drugs (ARVs) are provided free of charge, patients often have to pay out of pocket for other essential care such as laboratory tests and essential drugs including medicines for opportunistic infections and KS. The country is still recovering from an unprecedented large outbreak of Ebola virus disease that seriously impacted the already fragile health system and represented a major setback in the fight against HIV [12, 13]. The Ebola epidemic drew the attention of several international partners to support the global response to the crisis, and to aim to rebuild a more resilient health system. However, only a few international organisations are supporting the fight against HIV/AIDS.

In response to what may be considered as a neglected HIV epidemic in Guinea, Médecins Sans Frontières (MSF) has been working in the country since 1984, providing HIV services since the introduction of ART in 2003. Currently, in collaboration with the Ministry of Health, MSF provides support to over 8000 HIV patients (about a quarter of the national ART cohort). MSF has also been supporting chemotherapeutic care for patients with KS at DNH since 2012. Preliminary results in 2013 were encouraging, as 14 and 8 cases out of a selected 29 were reported to have demonstrated complete and partial remissions, respectively, after a round of 12 cycles of chemotherapy combined with ART. However, the study sample was small, subject to selection bias and thus inadequate to influence operational or programmatic decisions [14]. Since then, a cumulative number of over 250 patients had been enrolled for hospital care for KS in the unit, with unknown outcomes. We thus aimed to assess the outcomes of these patients in the short and medium term, as a proxy measure to evaluate the performance of the project implemented between 2012 and 2015.

## Methods

### Study site

The Donka National Hospital in Conakry is one of the two tertiary hospitals in the country, with its Dermatology Unit, being the sole referral centre for KS in the country.

### Diagnostic and therapeutic procedures

Patients diagnosed clinically with KS were referred to DNH, where they were seen by a dermatologist at the Dermatology Unit for specialised care. They were assessed clinically for evidence of KS, disease severity and comorbidities. Chest X-rays and abdominal scans were requested where necessary to look for visceral locations of the disease. An HIV test was performed for those with unknown status. Subsequently, patients were worked up for ART and chemotherapy. Baseline CD4 counts, complete blood counts, serum creatinine levels and liver enzyme assays were performed prior to initiation of ART and chemotherapy (and then serially as required during follow-up to monitor drug toxicities). The severity of KS was determined using the TIS staging system developed by the AIDS Clinical Trials Group (ACTG). The severity was based on the extent of the tumour (T), the status of the immune (I) system as measured by CD4 cell levels and the extent of involvement within the body or systemic (S) illness [15]. The choice for ARV regimen and antimitotic agents was tailored to a patient’s stage of disease, tolerability and special needs. In general, a combination of tenofovir (TDF), lamivudine (3TC) and efavirenz (EFV) was the preferred first line ART regimen. The primary chemotherapy regimen involved a combination of two cytotoxic agents, which were bleomycin (B) and vincristine (V). These agents were used with strict respect to MSF guidelines regarding safety, handling, preparation and administration of cytotoxic drugs [16]. Premedication with an analgesic, an antiemetic and antihistamine was recommended 30 min before administration of cytotoxics if a patient had experienced fever, chills or vomiting at the previous injection of cytotoxic. Bleomycin and Vincristine (BV) were administered in combination chemotherapy as 15 mg of intramuscular bleomycin followed by 2 mg of intravenous vincristine every 2 weeks for a series (or round) of 12 cycles. Bleomycin as a single agent was administered transiently in selected patients with severe systemic symptoms or with previous vincristine-induced haematological toxicity. As vincristine alone is of limited benefit, it was never used alone. Between cycles, patients were monitored both clinically, and through laboratory testing, for toxicity and treatment response. Treatment response was assessed using the ACTG criteria for complete or partial remission and stable or progressive disease [15]. Complete response (CR) was defined as absence of all evidence of disease and no appearance of new disease for a minimum of 4 weeks. Partial response (PR) was considered as a reduction by at least 50% in the number of all previously existing lesions, maintained for at least 4 weeks, with no new skin, oral or visceral lesions. An objective or overall response rate (ORR) was defined as the percentage of patients whose cancer shrank (PR) and/or disappeared (CR) after treatment. Stable disease (SD) was any response less than partial response, or where neither PR nor progressive disease (PD) criteria were met. PD was considered when there was more than 25% increase in one or more lesions, or appearance of new lesions. Depending on the response after the first round of chemotherapy, a second round may have been considered on a case by case basis. Psychosocial support was provided by trained lay staff or “expert HIV/KS patients” to help improve adherence to treatment and reduce the stigma associated with the disease.

### Study design

The study was a retrospective survival analysis of routinely collected clinical data of HIV-infected persons receiving ART and chemotherapy for KS between 2012 and 2015. We excluded 28 patients who were either enrolled in 2016, presented with endemic KS, or who had not started chemotherapy. Data were routinely collected from patients’ charts and then stored electronically in a spreadsheet. For the purpose of this study, we used the following variables: sociodemographics; date of HIV diagnosis; time since KS onset; ART starting date; ART regimen; date of enrolment into KS care; baseline CD4, haemoglobin and serum creatinine levels; site and stage of KS; chemotherapy initiation date and regimen; number of chemotherapy cycles; drug toxicities; treatment response; survival outcome; date of last clinical visit. The date of enrolment into KS care was set as the time of entry into the study (time 0). The patients were observed for a round of treatment cycles until a survival outcome or a treatment response event occurred. Survival outcomes were determined or censored on the 23rd of February 2016.

### Statistical analysis

Data analyses were performed using Stata® 14.2 (StataCorp LP, TX77845, USA). The dataset was checked for logical inconsistencies, illegal codes, omissions, and improbabilities by tabulating, summarising, describing and plotting variables. Missing observations were excluded where they constituted a small random proportion but were included if they were found to differ between categories of a given variable.

Our main outcomes of interest were survival, attrition from care and treatment response. Attrition from care was defined as a composite measure, consisting of the rate of occurrence of all-cause deaths and losses to follow-up (LTFU). A patient was classified as LTFU if there was no contact for 90 days or more after the last missed appointment for chemotherapy or ART refill. Retention in care was used to indicate the proportion of patients known to be alive and still receiving treatment at the time of the study. The rate of attrition as a time to event variable was measured as the number of deaths and LTFU expressed over the length of time in person-months. Missing two or more cycles of chemotherapy was considered as poor adherence to treatment. Treatment response was classified as CR, PR, ORR, SD or PD.

The explanatory variables examined were: gender; age; duration since KS onset; KS stage; CD4 counts; haemoglobin and creatinine levels; treatment cycles; treatment adherence.

Summary statistics were presented as proportions for categorical variables and as means [with standard deviations (sd)] for normally distributed continuous variables or medians [with Interquartile Ranges (IQR)] for skewed continuous variables. Pearson chi-squared tests or Fisher exact tests were used for small samples where appropriate to assess for differences in categorical variables between the two groups. A non-parametric test for trend was used to test for the median difference in categories for continuous variables. Kaplan-Meier survival curves were used to display the rate of attrition, while a log-rank test was used to assess for equality of survival functions. A univariable Cox regression model was set up to screen for factors associated with survival. Crude hazard ratios (HR) and their 95% confidence intervals (CI) were obtained. The *p*-values for hypotheses testing were calculated from likelihood ratio tests (LRT). Variables found to be associated with survival at a 5% confidence level were included in a multivariable Cox model. Backwards elimination based on *p*-values lower than 0.05 was used to retain variables independently associated with attrition. The corresponding adjusted hazard ratios (aHR), their 95% confidence intervals and *p*-values in the final model were reported [17]. The proportionality hazard assumption over time was assessed graphically using Aalen plots.

Similarly, factors associated with treatment response as a secondary outcome were screened using a multivariable logistic regression model. The corresponding adjusted odd ratios (aOR), their 95% confidence intervals and *p*-values were obtained. The performance of the model was assessed graphically using a receiver operating characteristic (ROC) curve.

## Results

### Study participants

A total of 225 patients with epidemic KS who presented for care after a median period of 12 months (IQR: 6–24) following disease onset and 6 months (IQR: 2–20) following HIV diagnosis, were included (Table 1). The sample consisted of 145 (64.4%) women, and their median age was 33 years (IQR: 27–43). Skin lesions were common to all patients, with associated oral lesions reported in 85 (37.8%), lymph node involvement in 8 (3.6%), eye involvement in two patients and peritoneum involvement in one patient. ACTG measurements though almost always incomplete were available for 191(84.9%) patients. Stage T1 disease was common at presentation (82.7%) and the median CD4 count was 231 cells/μl (IQR: 137–402) measured after a median duration of 6 months on ART (IQR: 0–14). The median baseline haemoglobin and creatinine levels were 10.7 (IQR: 10–12) and 77.9 (67.7–89.0), respectively. A drug history of chemotherapy was known for 24 (10.4%) patients.

**Table 1:** Descriptive characteristics of the study participants

Characteristics		Summary statistic
Enrolment per Calendar year, n (%)		
	2012	33 (14.7)
	2013	65 (28.9)
	2014	72 (32.0)
	2015	55 (24.4)
Age in years, median (IQR)		33 (27–43)
Gender, n (%)		
	Female	145 (64.4)
	Male	80 (35.6)
Duration between HIV diagnosis and enrolment in months, median (IQR)		6 (2–20)
Time before presentation in months, median (IQR)		12 (6–24)
History of KS treatment, n (%)		24 (10.7)
CD4 count in cells/μl, median (IQR)		231 (137–402)
Skin lesions, n (%)		
	Oedema	74 (32.8)
	> 20 lesions	75 (33.3)
	Raised lesions	125 (55.6)
	Skin thickening	122 (54.2)
Oral lesions, n (%)		85 (37.8)
Lymphadenopathy, n (%)		8 (3.6)
Visceral lesions, n (%)		3 (1.3)
Tumour stage T1, n (%)		186 (82.7)
Baseline haemoglobin level in g/dl, median (IQR)		10.7 (10–12)
Baseline creatininaemia in μmol/l, median (IQR)		77.9 (67.7–89.0)
Antiretroviral therapy regimen, n (%)		
	AZT-based	56 (24.9)
	D4T-based	7 (3.1)
	TDF-based	142 (63.1)
Duration on ART in months, median (IQR)		6 (0–14)
Chemotherapy cycles, median (IQR)		8 (2–12)
Severe adverse effects, n (%)		9 (4.0)
Poor adherence to chemotherapy, n (%)		19 (8.4)
Treatment response, n (%)		
	Complete remission	65 (28.9)
	Partial remission	53 (23.5)
	Stable disease	15 (6.7)
	Unknown response	92 (40.9)
Outcome, n (%)		
	Remain in care	105 (46.7)
	Lost to follow-up	92 (40.9)
	Dead	28 (12.4)

### Treatment response

After a median of 8 chemotherapy cycles (IQR: 2–12), complete response was observed in 65 (28.9%) and partial response in 53 (23.5%) patients, computing to an objective response rate of 52.4%. Stable disease and unknown response (UR) were reported respectively in 15 (6.7%) and 92 (40.9%) patients. All patients with an unknown response had dropped out from care and only underwent two cycles of chemotherapy on average. Severe cytotoxic events leading to treatment interruption occurred in 9 (4.0%) patients, including injection site skin necrosis in three, haematological toxicity (anaemia) in five and stomatitis in one patient. Overall treatment interruptions for a month or longer were reported in 19 (8.4%) patients.

**Fig. 1:**
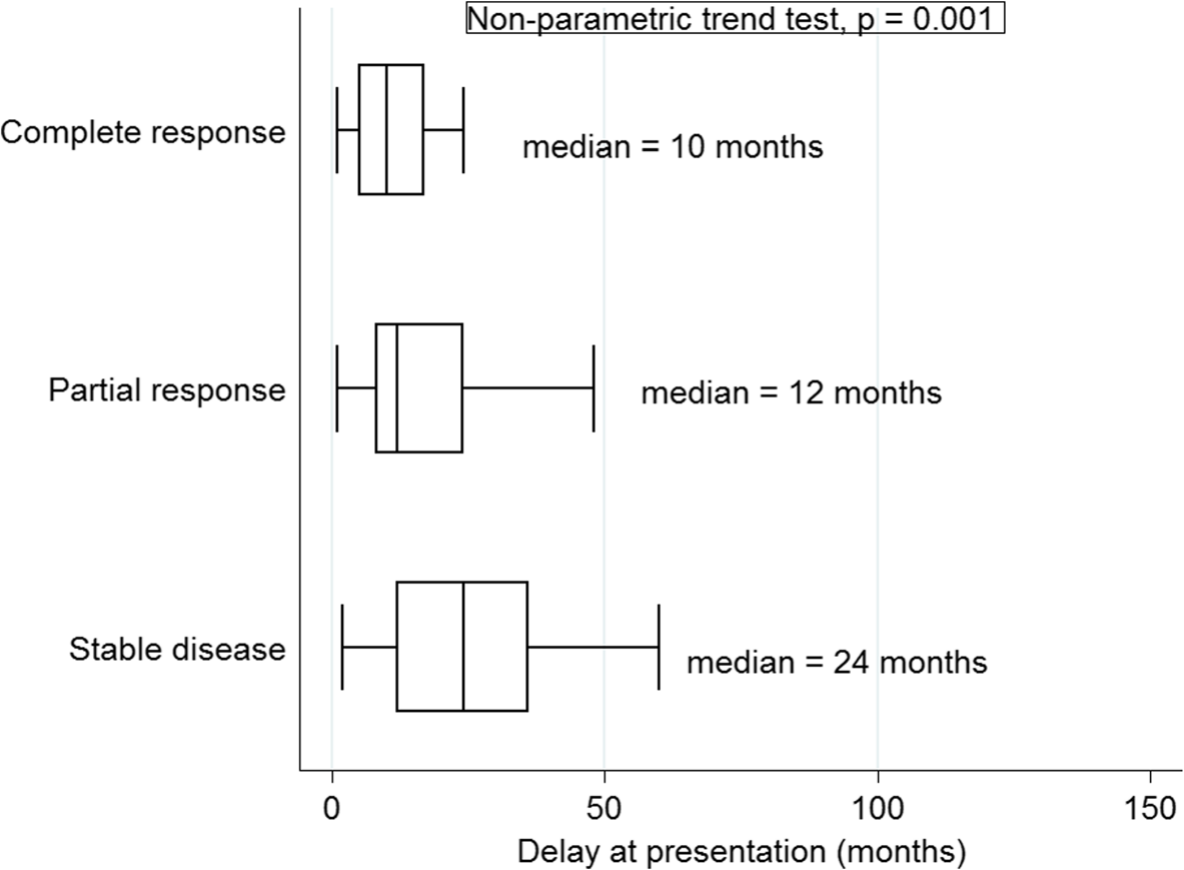
Treatment response and time since disease onset

Treatment response showed an inverse relationship with time since onset of KS: the shorter, the better the response. Median time lapses of 10, 12 and 24 months since KS onset were associated with CR, PR and SD respectively (trend test, *p* = 0.001; Fig. 1).

**Fig. 2:**
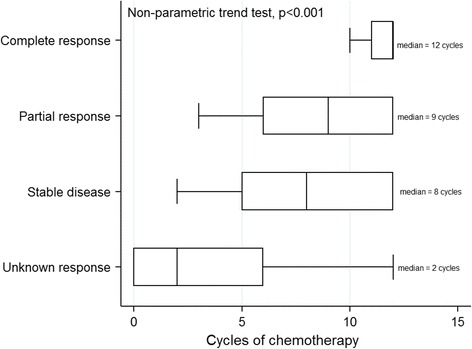
The relationship between duration of and response to chemotherapy

Treatment response also showed a direct relationship with the number of treatment cycles: more equalling better. Medians of 2, 8, 9, and 12 cycles of chemotherapy were associated with UR, SD, PR and CR, respectively, demonstrating a dose-response effect (trend test, *p* < 0.001; Fig. 2). The odds of achieving CR doubled after every additional cycle of chemotherapy (adjusted odd ratio, aOR = 2.09, 95% CI: 1.44–3.01) but were reduced by about two-thirds for every additional month of delay between onset of KS and enrolment into care (aOR = 0.31, 95% CI: 0.11–0.86). Complete response was seriously compromised in patients with woody skin oedema (aOR = 0.05, 95% CI: 0.01–0.38). Patients who were naïve to chemotherapy were significantly more likely to demonstrate a higher objective response rate than those previously exposed to chemotherapy (aOR = 4.72, 95% CI: 1.25–17.78; Table 2).

**Table 2:** Factors associated with treatment response in logistic regression models

Factor		Complete response rate N (%)	OR (95% CI)	*p*-value	aOR (95% CI)	*p*-value
Skin thickening						
	No	15 (62.5)	Reference		Reference	
	Yes	34 (40.5)	0.41 (0.16–1.04)	0.060	0.05 (0.01–0.38)	0.004
Each additional month delay at presentation			0.97 (0.95–0.99)	0.014	0.31 (0.11–0.86)	0.023
Each additional cycle of chemotherapy			1.42 (1.21–1.68)	<0.001	2.09 (1.44–3.01)	<0.001
Prior chemotherapy						
	Exposed	14 (73.7)	Reference		Reference	
	Naive	103 (92.0)	4.09 (1.20–13.95)	<0.001	4.72 (1.25–17.78)	0.022

### Survival outcomes

**Fig. 3:**
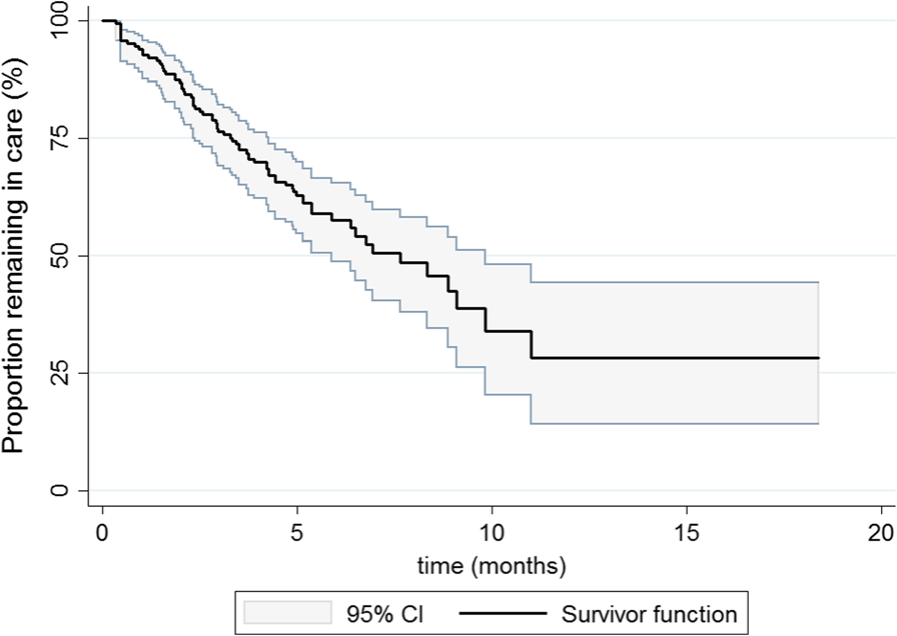
Overall Kaplan-Meier survival curve

**Fig. 4:**
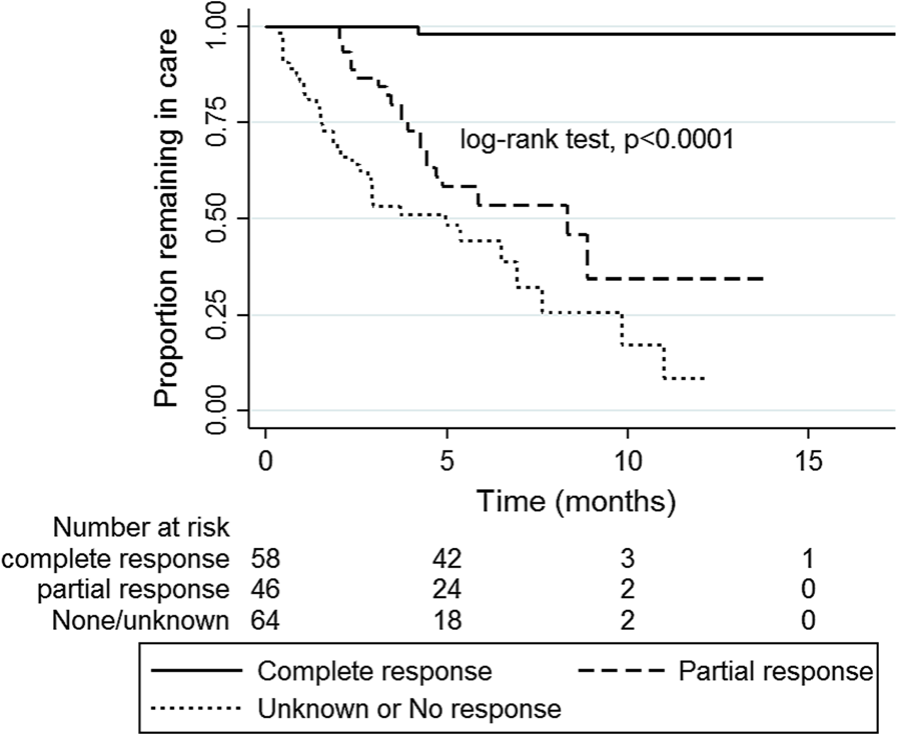
Survival curves according to treatment response

After a total observation period of 812 person -months, 105 (46.7%) patients were still in care while 92 (40.9%) patients were lost to follow-up and 28 (12.4%) patients were confirmed dead. The overall median survival time was 7.6 months (95% CI: 5.9–9.8; Fig. 3). Retention or survival in care depended on the nature of the treatment response (Fig. 4). Mortality and defaulting on treatment were reduced by 22% for every cycle of chemotherapy (aHR = 0.78, 95% CI: 0.71–0.84) and were about 20 times lower in those with complete remission, compared with those with partial or no response (aHR = 0.05, 95% CI: 0.007–0.43; Table 3).

**Table 3:** Factors associated with attrition from care in a Cox model

Factor		Attrition N (Rate per 1000 person-months)	HR (95% CI)	*p*-value	aHR (95% CI)	*p*-value
Each additional cycle of chemotherapy			0.75 (0.70–0.80)	<0.001	0.78 (0.71–0.84)	<0.001
Treatment response						
	Complete	1 (2.8)	Reference		Reference	
	Partial	21 (88.3)	30.53 (4.11–227.13)	0.001	19.56 (2.61–146.91)	0.001
	Unknown or No response	8 (160.0)	57.19 (7.8–417.9)	<0.001	12.83 (1.61–102.19)	0.016

## Discussion

We aimed to evaluate the outcomes of patients treated for KS in a sub-Saharan African context, where a paucity of information on KS therapy exists. This is partly attributed to the scarcity of resources available for provision of KS treatment [18]. The study indicated that more patients were accessing treatment for KS than ever before and about one out of two patients had responded objectively to treatment. However, poor outcomes were also common as a result of late presentation and dropping out of care.

The number of patients seeking hospital care for KS at the Dermatology Unit of DNH has increased exponentially within the last few years. Between 2000 and 2009, about 134 patients were documented to have been enrolled in care [11], but we identified over 250 in the last four years. Elsewhere, the International Epidemiologic Databases to Evaluate AIDS (IeDEA) Consortia in the period 2009–2012 identified 677 cases from Kenya, 172 from Uganda, 57 from Nigeria, 67 from Cameroon and 355 from Malawi [3]. While East Africa is traditionally known as the hotbed of KS in the world [19, 20], the rising burden of KS in Guinea is consistent with the prevalent situation in the region where KS is the most commonly diagnosed malignancy associated with HIV. This is especially the case in West and Central Africa, where ART coverage remains low [6, 18, 21]. In Guinea, an increased ascertainment of KS and referral by physicians may be a contributing factor, together with an increased access to KS treatment, due to the support provided by MSF. Still, most patients presented not only with advanced HIV disease but also with an advanced KS, probably due to poor awareness or reliance on traditional medicine. The distance to travel to the capital city for those in remote areas might not only be a factor for late presentation, but also a contributor to early drop out from care and an obstacle for accessing treatment. This double burden of HIV/KS explains the high rates of attrition from care. Most late presenters could only benefit from palliative care to improve their quality of life. Under such circumstances, the goal of treatment may not be to achieve a high complete response rate but a high objective response rate (ORR = CR + PR), which is a surrogate endpoint for survival [22]. Thus, attaining an ORR of 52.4% in this cohort may be considered a satisfactory result for the patients, care givers and the programme. However, due to issues related to under- or overestimation of treatment effects, an ORR may not fully capture the net benefits of survival and should thus be interpreted with some caution [23]. We performed survival analyses based on Kaplan-Meier curves and Cox regression to demonstrate a correlation between treatment response and survival and, we observed that complete and partial responders lived longer than unknown or non-responders. Therefore, ORR could be a reliable marker of survival in advanced KS. This corroborates with findings from an earlier study on metastatic breast cancer [22]. We believe that defaulters with an unknown response and unknown vital status were probably non-responders and had died. This is because on one hand, they received only a median of two cycles of chemotherapy (treatment response was dose-dependent and vice versa); and on the other hand, the rate of attrition in the HIV cohort in Guinea at the same time was about 25% overall, which is much lower than the rate observed by the KS patients. Conversely, it was possible that some defaulters had responded at least partially to treatment before dropping out from care and thus resulting to an underestimation of the true overall response rate. This uncertainty might also explain the lack of precision (wide 95% CI) around the survival estimates between partial responders and non-responders (Table 3). Our overall findings seem to correlate with results from most other African countries where loss to follow up is very high and generally very poor KS treatment responses, though excellent outcomes have also been described in some African settings [3, 4, 21, 24]. Therefore, causes of loss to follow up should be investigated and strategies to mitigate them implemented.

The presence of woody oedema was a predictor of a poor treatment response. This form of skin thickening has been linked to long-standing skin oedema, and the resulting reactional inflammatory fibrosis is indicative of advanced disease or stage T1 [15]. Poor treatment outcomes have been reported in children and adolescents who presented with skin oedema in Malawi [25]. Clinicians need to identify KS at an early stage by raising their index of suspicion amongst other differential diagnoses in the presence of skin oedema, especially in patients infected with HIV.

Previous exposure to chemotherapy was also predictive of poor treatment response. It is very likely that this group of patients were restarting a treatment regimen that failed them earlier on, given that the country had few options for KS treatment. We recommend such patients to be candidates for second line therapy, which is now available in the form of pegylated liposomal doxorubicin (PLD). Although it is not certain that this will be successful because there have been no significant differences between various chemotherapy regimens in the treatment of severe or progressive Kaposi’s sarcoma in HIV-infected adults [26]. However, it is reasonable alternative to remove patients from a failed regimen and attempt other options.

Our study had some limitations. The diagnosis of KS was clinical rather than pathologic; while clinical diagnosis is very typical of SSA settings, and probably reasonably accurate in the correct context of HIV with classical skin lesions, it is clearly inaccurate with a recent study finding only 77% positive predictive value for clinically suspected KS compared to final histologic diagnosis in some East African HIV clinics [19]. The ACTG staging was incomplete and thus we could not account for disease severity in our attrition analyses. Treatment response was not ascertained for patients who dropped out of care, although we assumed with some degree of uncertainty that they were likely to have a progressive disease. This assumption must have led to an underestimation of the overall response rate. Treatment regimens varied between and within patients from one cycle to another, and it was therefore difficult to assess the effect of a single or combination therapy. The observed dose-response effect of chemotherapy appeared plausible but not causal because the temporal sequence of treatment duration and response could not be determined given the retrospective and incomplete nature of our data. A better response could as well have led to longer chemotherapy durations or cycles (and vice versa).

## Conclusions

In conclusion, access to KS care has expanded in Guinea, and the overall response rate to KS treatment has been satisfactory. Poor outcomes are not uncommon and are largely due to late presentation and defaulting on treatment. Efforts towards early diagnosis and adherence to a complete series of chemotherapy are mandatory for optimising outcomes. Newer drugs may be required for patients previously exposed to chemotherapy.

## Additional file

Additional file 1: S1. Dataset chemotherapy KS. (XLSX 49 kb)

## Abbreviations

ACTG: AIDS Clinical Trials Group
aHR: adjusted Hazard Ratio
AIDS: Acquired Immune Deficiency Syndrome
aOR: adjusted Odd Ratio
ART: Antiretroviral Therapy
ARV: Antiretroviral drug
CD: Cluster Designation
CI: Confidence Interval
CR: Complete Response
DNH: Donka National Hospital
HIV: Human Immunodeficiency Virus
IQR: Interquartile Range
KS: Kaposi Sarcoma
MSF: Médecins Sans Frontières
n: number of observations
ORR: Overall Response Rate
PD: Progressive Disease
PR: Partial Response
SD: Stable Disease
STD: Sexually Transmitted Disease
UR: Unknown Response
